# Altered Spontaneous Brain Activity Related to Neurologic Dysfunction in Patients With Cerebral Small Vessel Disease

**DOI:** 10.3389/fnagi.2021.731585

**Published:** 2021-12-17

**Authors:** Mengmeng Feng, Hongwei Wen, Haotian Xin, Nan Zhang, Changhu Liang, Lingfei Guo

**Affiliations:** ^1^Department of Radiology, Cheeloo College of Medicine, Shandong Provincial Hospital, Shandong University, Jinan, China; ^2^Key Laboratory of Cognition and Personality, Ministry of Education, Chongqing, China; ^3^School of Psychology, Southwest University, Chongqing, China; ^4^Department of Radiology, Shandong Provincial Hospital Affiliated to Shandong First Medical University, Jinan, China

**Keywords:** cerebral small vessel disease, cerebral microbleeds, blood oxygen level-dependent, low-frequency fluctuation, regional homogeneity

## Abstract

Cerebral small vessel disease (CSVD) encompasses several diseases affecting the small arteries, arterioles, venules, and capillaries of the brain and refers to several pathological processes and etiologies. Neuroimaging is considered the gold standard for detecting CSVD, which can present diverse features on MRI. Cerebral microbleeds (CMBs) in CSVD have been demonstrated to play a synergistic role in both cerebrovascular and neurodegenerative pathology. Considering previous studies on brain structural abnormalities in CSVD, in the present study, we aimed to explore altered spontaneous brain activity among CSVD patients using amplitude of low-frequency fluctuation (ALFF), fractional ALFF (fALFF) and regional homogeneity (ReHo) methods based on resting-state functional MRI. In this study, we recruited 24 CSVD patients with CMBs (CSVD-c), 42 CSVD patients without CMBs (CSVD-n) and 36 healthy controls from outpatient clinics in Shandong Provincial Hospital affiliated to Shandong First Medical University between September 2018 and June 2019. All subjects underwent 3-T MRI, including blood oxygen level-dependent (BOLD) and susceptibility-weighted imaging (SWI). Anatomic structures were segmented, ALFF/fALFF values were calculated, and ReHo maps were generated. Further statistical analysis was applied to study the difference in ALFF/fALFF/ReHo among the three groups and the association between ALFF/fALFF/ReHo changes in different brain regions and clinical characteristics. Twenty-four CSVD-c patients (age: 67.54 ± 6.00 years, 10 females), 42 CSVD-n patients (age: 66.33 ± 5.25 years, 22 females) and 36 healthy subjects (age: 64.14 ± 8.57 years, 19 females) were evaluated. Compared with controls, the CSVD-c group showed significantly increased ALFF values in the right insula, putamen and left precuneus; decreased fALFF values in the right precentral gyrus and postcentral gyrus; and increased ReHo values in the left precuneus, fusiform gyrus, right supplementary motor area (SMA), and superior frontal gyrus. Notably, the mean ALFF values of the right insula and putamen were not only significantly related to all clinical parameters but also demonstrated the best performance in Receiver Operating Characteristic (ROC) curve analysis. These findings reveal CSVD-c patients have dysfunctions in the default mode network, sensorimotor network and frontoparietal network, which may implicate the underlying neurophysiological mechanisms of intrinsic brain activity. The correlation between altered spontaneous neuronal activity and clinical parameters provides early useful diagnostic biomarkers for CSVD.

## Introduction

Cerebral small vessel disease (CSVD) encompasses multiple pathological processes and etiologies that affect small cerebral blood vessels, such as arteries, arterioles, capillaries, and small veins of the brain (Cuadrado-Godia et al., 2018). The pathogenesis of CSVD is not well known, but blood-brain barrier (BBB) leakage seems to be a common and primeval mechanism (Farrall and Wardlaw, 2009). The characteristic MRI features of CSVD include cerebral microbleeds (CMBs), white matter (WM) hyperintensities (WMHs), recent small subcortical infarcts, lacunes, perivascular spaces (PVSs), and brain atrophy (Qiu et al., 2018). Stroke, cognitive decline, dementia, psychiatric disorders, and gait disturbances caused by CSVD have been well demonstrated in elderly individuals. Pertinently, early detection of CSVD is crucial.

CMBs are one of the three factors that affect the total CSVD score. Regardless of the size, location and number of microbleeds, the presence or absence of microbleeds can directly affect the CSVD score (Amin Al Olama et al., 2020), so microbleeds are a more important feature in CSVD patients. CMBs are most easily observed in T2-weighted gradient-recalled echo (GRE) or susceptibility-weighted imaging (SWI) sequences, usually as round or ovoid small hypointense areas with associated blooms seen on GRE scans (Shi and Wardlaw, 2016), which is not easy to see on CT scans, T2 fluid-attenuated inversion recovery images, or T1-weighted images. CMBs are related to decreases in executive function, information processing, memory function, and movement speed (Moulin et al., 2016). The prevalence of CMBs was higher in patients with cognitive decline than in the general population (Lim et al., 2020). Therefore, we divided the subjects into three subgroups: CSVD with CMBs (CSVD-c), CSVD without CMBs (CSVD-n) and control; these groups were evaluated to further illustrate the changes in brain function in CSVD-c patients.

In recent years, a number of studies have illustrated the structural changes in patients with CSVD. In CSVD patients, the bilateral dorsolateral prefrontal, parietal and posterosuperior temporal cortices—especially in the occipital and sensorimotor cortices—became thinner (Lambert et al., 2015). Gait disorder caused by CSVD is related to the volume of periventricular WMH and fractional anisotropy (FA) in the frontal and parietal regions (Kim et al., 2016). The volume of gray matter (GM) in some subregions of the frontal lobes, the parahippocampal gyrus, the temporal lobes, hippocampus and thalamus was reduced in CSVD patients with high WMH scores (Wang et al., 2020). Taken together, several studies have demonstrated that cortical thinning or GM volume reductions occur in CSVD patients in different brain areas.

In addition to previous structural imaging studies of CSVD, ongoing neuroimaging studies have shown changes in brain function and functional connectivity in patients with CSVD. The functional connectivity (FC) of the right thalamus, hippocampus, and precuneus was lower in CSVD patients, and the FC of the right inferior parietal lobule was higher in CSVD patients with cognitive impairment group (Liu et al., 2019). The anterior cingulate cortex and the supplementary motor area (SMA) with medial prefrontal cortex showed lower FC in CSVD subjects with cognitive impairment (Zhou et al., 2016). In CSVD patients with gait disorder, fractional ALFF (fALFF) values decreased in areas mainly located in the prefrontal network and sensorimotor network, such as the left superior parietal gyrus and the left SMA (SMA.L), while fALFF values in the left precuneus, the left caudate and the right inferior frontal gyrus (orbital part) increased (Zhou et al., 2020).

As new magnetic resonance technology has received increasing attention, resting-state functional magnetic resonance imaging (rs-fMRI), a non-invasive technique, has been widely applied to describe the internal funcetional patterns of the brain, and it is a supporting technology for the study of brain functional organizations and patterns at present. Amplitud of low-frequency fluctuation (ALFF), fALFF and regional homogeneity (ReHo) are three potentially useful rs-fMRI tools for quantification of neural activity based on blood oxygen level-dependent (BOLD) signals. The ALFF method detects the total power spectrum within the range between 0.01 and 0.10 Hz and ALFF values positively correlate with alterations in spontaneous neural activity (Yu-Feng et al., 2007; Zou et al., 2008). However, physiological noise is difficult to eliminate, and the fALFF technique has been suggested to use to calculate the ratio of the low-frequency power spectrum to that of the whole frequency range (Zou et al., 2008). Both ALFF and fALFF approaches have been proven to exhibit greater test-retest reliability, especially in GM (Zuo et al., 2010). ReHo is a data-driven approach based on voxel-wise analysis, which reflects the similarities and coherence of spontaneous low-frequency (<0.08 Hz) signal fluctuations throughout the brain (Dai et al., 2014). Recently, these methods have been widely used to explore brain diseases with potential functional alterations, such as depression (Yu et al., 2019), Alzheimer’s disease (Cheng et al., 2019), and Tourette syndrome (Liu et al., 2017). Accordingly, the combination of the three approaches may provide more detailed information about intrinsic activity across the whole brain.

In the present study, we investigated the abnormal intensity of neural activity via ALFF/fALFF analysis in CSVD patients. Based on previous studies, we hypothesized that (1) CSVD-c patients would show significant brain functional changes in several brain regions compared with controls and CSVD-n patients, (2) alterations in spontaneous brain activity would be related to clinical parameters in CSVD patients, and (3) abnormal spontaneous activity patterns might be utilized as diagnostic neuroimaging biomarkers to distinguish CSVD-c patients from controls. We aim to take a crucial step toward identifying spontaneous brain activity abnormalities in CSVD-c patients and toward providing potential targets to improve the present understanding of and treatment strategies for this neurologic disorder.

## Materials and Methods

### Subjects

Twenty-four CSVD-c patients (age: 67.54 ± 6.00 years, 10 females) and 42 CSVD-n patients (age: 66.33 ± 5.25 years, 22 females) were recruited from outpatient clinics in Shandong Provincial Hospital affiliated to Shandong First Medical University between September 2018 and June 2019. We also included 36 age- [*p* = 0.646, analysis of covariance (ANCOVA) test] and sex- (*p* = 0.646, chi-square test) matched healthy subjects (age: 64.14 ± 8.57 years, 19 female) in our study. The severity of CSVD was assessed according to amended SVD score (Amin Al Olama et al., 2020). CMBs were scored on the basis of absence or presence, not location or quantity (Debette et al., 2019). In this study, 7 CSVD-c patients belong to lobar CMBs group and 17 CSVD-c patients belong to deep CMBs group (Liu et al., 2020). WMHs were graded using Fazekas scale (0–3) (Fazekas et al., 1987), and the number of lacunes was graded from 0 to 3 (0 = none, 1 = 1–2, 2 = 3–5, 3 = >5) (Amin Al Olama et al., 2020).

The exclusion criteria included: (1) a history of psychiatric or neurological illness; (2) a history of brain trauma, epilepsy, cerebral apoplexy, brain tumors and depression; (3) a history of thrombolysis; (4) a history of alcohol or substance abuse; (5) the presence of heart, liver, and kidney damage; (6) acute complications of Type 2 diabetes and severe hypertension; (7) severely impaired visual and auditory functions. All participants are voluntary and signed an informed consent prior to the start of the study.

### Cognitive Assessment

All participants underwent the neuropsychological scale. Cognitive functions were globally assessed using the Montreal Cognitive Assessment (MoCA) Beijing version,^1^ which is a one-page 30-point test administered in 10 min (Nasreddine et al., 2005; Bergeron et al., 2017; Tian et al., 2020). The optimal cutoff for detecting cognitive impairment points was 13/14 for illiterate individuals, 19/20 for individuals with 1–6 years of education, and 24/25 for individuals with 7 or more years of education (Lu et al., 2011). One point is added if the education years is less than 12. Rey auditory verbal learning test (AVLT), the symbol digit modalities test (SDMT), the trail-making test (TMT) and the Stroop color-word test (SCWT) (Scarpina and Tagini, 2017) were also performed to assess verbal memory ability (Putcha et al., 2019), attention and information processing speed (Benedict et al., 2017) and visual search and motor coordination (Wei et al., 2018). The test implementer was professionally trained and qualified, who has no knowledge of the subject grouping.

### Image Acquisition

MRI scanning was performed with a MAGNETOM 3-Tesla MR scanner (Skyra, Siemens Healthcare, Erlangen, Germany). Before the scan, all participants were to maintain normal respiration and a normal heart rate. All participants were required to be awake and quietly breathe until the end of the scan. The scanner parameters for BOLD were repetition time/echo time (TR/TE) = 1,500/30ms, slice thickness = 3mm, field of view (FOV) = 24 × 24cm^2^; the parameters for SWI were TR/TE = 27/20ms, slice thickness = 1.5 mm, and FOV = 22 × 22cm^2^. A three-dimensional T1-weighted (T1W) magnetization-prepared rapid acquisition gradient-echo sequence (TR/TE = 2300/2.32ms, slice thickness = 0.9 mm, FOV = 24 × 24cm^2^) was performed for anatomic reference. The patients also underwent T2-weighted, T2 fluid-attenuated inversion recovery, quantitative susceptibility mapping and arterial spin labeling sequences.

### Data Preprocessing

Resting-state fMRI data reprocessing was performed using statistical parametric mapping (SPM8) and Data Processing & Analysis for Resting-state Brain Imaging (DPABI Version 2.1^2^). The first 10 image volumes of functional images were removed for signal equilibrium and subject adaptation to scanning noise. Then, functional images were corrected for time offsets between slices and geometrical displacements due to head motion. We further calculated the mean frame-wise displacement (FD) to measure voxel-wise differences in motion in its derivation (Jenkinson et al., 2002). None of the participants were excluded based on the exclusion criteria of maximum head motion of 3.0 mm and 3.0 degrees, with mean FD > 0.2 mm. The T1-weighted images were co-registered to the average functional images and then segmented into WM, GM, and cerebrospinal fluid (CSF) by using the *New Segment* tool in DPABI. We removed linear trends and regressed out several nuisance signals from the time course of each voxel, including 24-parameter head-motion profiles (Friston et al., 1996; Yan et al., 2013) and mean WM and CSF time series within the respective brain masks derived from prior probability maps in SPM8 (threshold = 0.8). All the corrected functional data were then normalized by the *DARTEL* (Ashburner, 2007) tool to Montreal Neurological Institute (MNI) space using an optimum 12-parameter affine transformation and non-linear deformations; then, they were resampled to a 3-mm isotropic resolution.

### Measurement of Amplitude of Low-Frequency Fluctuation/Fractional Amplitude of Low-Frequency Fluctuation and Regional Homogeneity

To calculate ALFF, we first performed spatial smoothing on the resampled images with a 4mm full width at half maximum (FWHM) Gaussian kernel. Then, we converted the smoothed signal of each voxel from the time domain to the frequency domain via fast Fourier transform (FFT) to obtain the power spectrum. This power spectrum (frequency range: 0–0.25Hz) was square-rooted at each frequency and then averaged across 0.01–0.08 Hz at each voxel; the result was regarded as the ALFF (Yu-Feng et al., 2007). Then, a ratio of the sum of the amplitude within the low-frequency band (i.e., ALFF) to that of the entire frequency band (0–0.25Hz) was computed as the fALFF value (Zou et al., 2008). The ALFF/fALFF of each voxel was divided by the global mean ALFF/fALFF for standardization purposes, and the mean ALFF/fALFF was obtained as a parameter for further statistical comparison and analysis.

ReHo maps were generated before spatial smoothing. After normalization, bandpass filtering (0.01–0.08 Hz) was performed on the normalized images to reduce the effects of low-frequency drift and high-frequency physiological noise. ReHo maps were conducted by calculating the Kendall coefficient of concordance (KCC) for a given voxel time series with those of its nearest 27 neighbors (Zang et al., 2004). For standardization purposes, the ReHo value of each voxel was divided by the whole brain mean ReHo value; then, the standardized ReHo map was smoothed with a 4mm FWHM Gaussian kernel (i.e., smReHo). The smReHo map was obtained as the ReHo parameter for further statistical comparison and analysis.

### Statistical Analysis

Further statistical analysis was performed based on a 90% group mask (meaning 90% of subjects had this voxel) generated in the *DPABI* toolbox to detect group differences. To compare ALFF/fALFF and identify abnormalities among the three groups, one-way ANCOVA was performed with age, sex and mean FD as covariates. For the *post hoc* test, the least-significant difference (LSD) method was applied, and the corrected *p*-values for comparing group means of any pairs were calculated (Yan et al., 2016). Then, the p maps were converted to Z maps, and using the Z maps, we performed Gaussian random field (GRF) correction (Bansal and Peterson, 2018) to correct for multiple comparisons. The statistical thresholds were set at *p* < 0.001 at the voxel level and *p* < 0.05 at the cluster level (two-tailed) in the *DPAB*I toolbox (Yan et al., 2016). All coordinates were reported in MNI space. Brain regions with significant intergroup differences in ALFF/fALFF/ReHo were defined as regions of interest (ROIs), and the mean ALFF/fALFF/ReHo values of these ROIs were extracted from CSVD patients. Pearson’s correlations between ALFF/fALFF/ReHo values and clinical parameters were calculated using SPSS Version 24.0 (SPSS Inc., Chicago, IL, United States), and the significance threshold was set to *p* < 0.05.

### Receiver Operating Characteristic Curves Analysis

The mean ALFF/fALFF/ReHo values of significantly altered brain clusters were extracted and used for analysis of the Receiver Operating Characteristic (ROC) curves using MedCalc Statistical Software.^3^ To summarize the overall diagnostic ability of the tests, we computed the maximum Youden index (sensitivity + specificity – 1) (Fluss et al., 2005) and the corresponding sensitivity, specificity and 95% confidence intervals (CIs) for each cluster.

## Results

### Demographic and Clinical Characteristic

The demographic and clinical characteristics of each group are summarized in Table 1. Hypertension and hyperlipidemia were more often present in CSVD patients compared with others. The CSVD-c group had significantly lower Montreal Cognitive Assessment (MoCA), Auditory Verbal Learning Test (AVLT), and Symbol Digit Modalities Test (SDMT) scores and significantly higher Stroop Color-Word Test (SCWT) and Trail-Making Test (TMT) scores than the other groups. No significant differences were found in age, sex, diabetes or mean FD between the patient and control groups. The prevalence of WMHs did not differ significantly between the two CSVD groups, however, the prevalence of lacunes was higher in CSVD-c group as compared with CSVD-n group.

**TABLE 1:** Demographic and clinical characteristics of CSVD patients and controls.

Characteristics	CSVD patients with CMBs	CSVD patients without CMBs	HCs	*P*-value
Sex	14 M/10 F	20 M/22 F	17 M/19 F	0.646^χ2^
Age (y)	67.54 ± 6.00	66.33 ± 5.25	64.14 ± 8.57	0.140^a^
Hypertension, n (%)	22 (92)	36 (86)	12 (33)	<0.001^χ2^
Diabetes, n (%)	9 (38)	8 (19)	4 (11)	0.044^χ2^
Hyperlipidemia, n (%)	15 (63)	13 (31)	5 (14)	<0.001^χ2^
MoCA	25.52 ± 2.82	27.55 ± 0.89	28.66 ± 0.87	<0.001^a^
AVLT	54.48 ± 16.91	64.93 ± 9.55	68.14 ± 8.47	<0.001^a^
SDMT	23.26 ± 10.94	30.22 ± 9.05	39.63 ± 14.32	<0.001^a^
SCWT	187.13 ± 71.17	145.90 ± 27.55	134.83 ± 38.12	<0.001^a^
TMT-A + B	346.17 ± 175.25	259.78 ± 76.34	213.26 ± 101.20	<0.001^a^
FD_Jenkinson	0.13 ± 0.07	0.13 ± 0.08	0.11 ± 0.04	0.339^a^
WMHs	1.96 ± 0.91	1.57 ± 0.70		0.057^b^
Lacunes	0.88 ± 0.99	0.05 ± 0.22		<0.001^b^

*CSVD, cerebral small vessel disease; CSVD-c, CSVD with CMBs; CSVD-n, CSVD without CMBs; χ^2^, chi-square test; ^a^ANCOVA test; MoCA, Montreal Cognitive Assessment; AVLT, sum of Rey Auditory Verbal Learning Test (N1-7); SDMT, Symbol Digit Modalities Test; SCWT, sum of Stroop Color-Word Test (Stroop 1-3); TMT, Trail-Making Test; TMT-A + B, sum of TMT-A and TMT-B; FD_Jenkinson, frame-wise displacement (Jenkinson et al., 2002); WMHs, white matter hyperintensities; ^b^Wilcoxon test.*

### Amplitude of Low-Frequency Fluctuation Results

**FIGURE 1:**
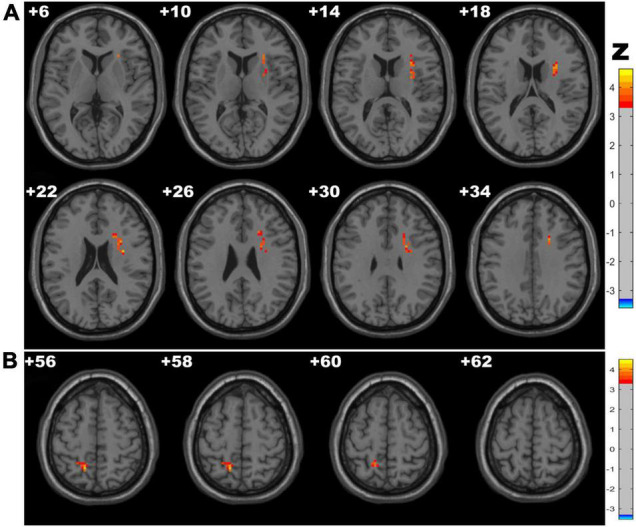
Clusters with significantly altered ALFF values among groups [ANOVA and the least-significant difference (LSD) *post hoc* test with Gaussian random field (GRF) correction, voxel-level *p* < 0.001, cluster-level *p* < 0.05]. The red-yellow areas denote higher ALFF values in the CSVD-c group than in the **(A)** control groups or **(B)** CSVD-n group.

The CSVD-c group showed significantly increased Amplitude of Low-Frequency Fluctuation (ALFF) values in the clusters of the right insula, putamen and left precuneus compared with the control group and showed significantly increased ALFF values in the clusters of the left precuneus compared with the CSVD-n group. No significant difference was found between the CSVD-n group and the control group. The details are presented in Table 2 and Figure 1.

**TABLE 2:** Significant differences in ALFF values among groups (ANOVA and LSD *post hoc* test with GRF correction, voxel-level *p* < 0.001, cluster-level *p* < 0.05).

Condition	Brain regions	Cluster size	Z-score of the peak voxel	MNI coordinates of the peak voxel		
				*x*	*y*	*z*
CSVD-c > HC	Right insula/putamen	94	4.47	27	12	24
	Left precuneus	29	4.22	–15	–54	57
CSVD-c > CSVD-n	Left precuneus	16	4.28	–15	–54	57

*MNI, Montreal Neurological Institute.*

### Fractional Amplitude of Low-Frequency Fluctuation Results

**FIGURE 2:**
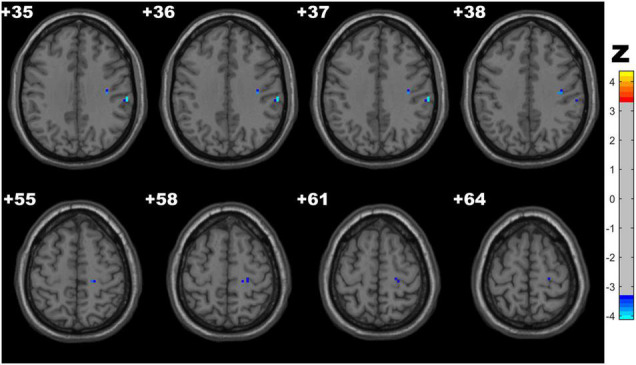
Clusters with significantly altered fALFF values among groups (ANOVA and LSD *post hoc* test with GRF correction, voxel-level *p* < 0.001, cluster-level *p* < 0.05). The blue areas denote lower fALFF values in the CSVD-c group than in the control group.

Compared with the control group, the CSVD-c group showed significantly lower fALFF values in the clusters of the right precentral gyrus and postcentral gyrus. No significant difference was found between the other pair-wise groups. The details are presented in Table 3 and Figure 2.

**TABLE 3:** Significant differences in fALFF values among groups (ANOVA and LSD *post hoc* test with GRF correction, voxel-level *p* < 0.001, cluster-level *p* < 0.05).

Condition	Brain regions	Cluster size	Z-score of the peak voxel	MNI coordinates of the peak voxel		
				*x*	*y*	*z*
CSVD-c < HC	Right precentral gyrus	13	–4.12	36	–18	39
	Right postcentral gyrus	4	–4.12	60	–24	36

### Regional Homogeneity Results

**FIGURE 3:**
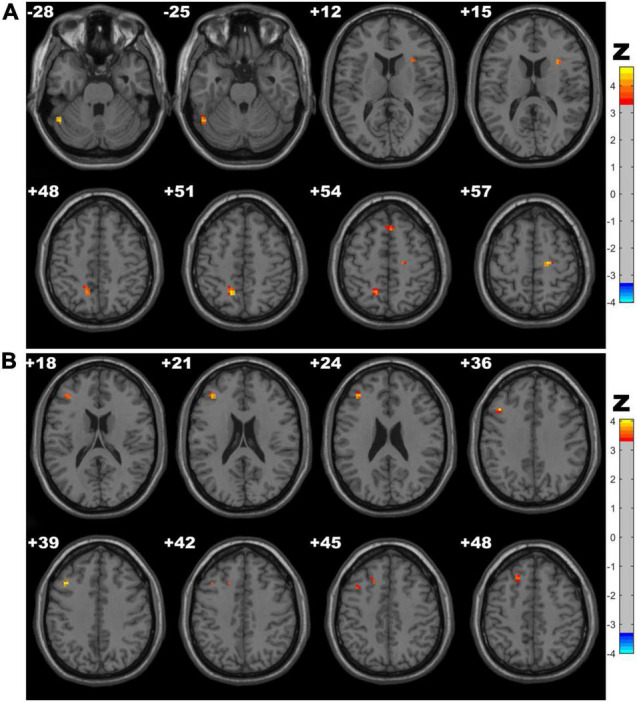
Clusters with significantly altered ReHo values among groups (ANOVA and LSD *post hoc* test with GRF correction, voxel-level *p* < 0.001, cluster-level *p* < 0.05). The red-yellow areas denote higher ReHo values in the CSVD-c group than in the **(A)** control groups or **(B)** CSVD-n group.

CSVD-c group showed significantly increased ReHo in the clusters of the left precuneus, fusiform gyrus and right SMA, superior frontal gyrus compared with controls, and showed significantly increased ReHo in the clusters of the left middle frontal gyrus, superior frontal gyrus and right SMA compared with controls. No significant difference was found between the CSVD-n group and the control group. The details are presented in Table 4 and Figure 3.

**TABLE 4:** Significant differences in ReHo values among groups (ANOVA and LSD *post hoc* test with GRF correction, voxel-level *p* < 0.001, cluster-level *p* < 0.05).

Condition	Brain regions	Cluster size	Z-score of the peak voxel	MNI coordinates of the peak voxel		
				*x*	*y*	*z*
CSVD-c > HC	Left precuneus	29	4.70	–15	–54	51
	Right supplementary motor area	19	4.68	18	–18	57
	Left fusiform gyrus	12	4.59	–48	–57	–27
	Right superior frontal gyrus	17	4.12	3	24	54
CSVD-c > CSVD-n	Left middle frontal gyrus	30	4.06	–42	21	39
	Left superior frontal gyrus	10	3.68	–21	27	48
	Right supplementary motor area	10	3.68	12	3	63

### Receiver Operating Characteristic Curves Analysis

**FIGURE 4:**
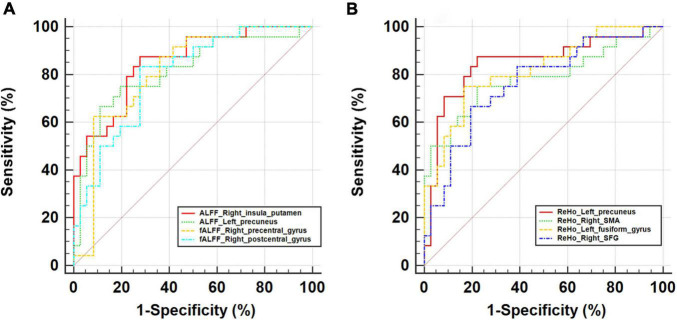
ROC curve for altered brain clusters relevant to **(A)** ALFF/fALFF and **(B)** ReHo that distinguish CSVD-c patients from controls. ALFF, amplitude of low-frequency fluctuation; fALFF, fractional ALFF; ReHo, regional homogeneity; SMA, supplementary motor area; SFG, superior frontal gyrus.

As ROC curve analysis, which distinguished CSVD-c patients from controls, showed, the mean ALFF value of the right insula/putamen achieved the best classification performance considering sensitivity, area under the ROC curve (AUC) and 95% CIs. All the altered brain clusters achieved a significance level of *p* < 0.001 for AUC, indicating that these findings are significant and potentially useful diagnostic biomarkers. The details are presented in Table 5 and Figure 4.

**TABLE 5:** The statistics of ROC curve analysis for altered brain clusters that distinguish CSVD-c patients from controls.

Clusters	SEN	SPE	AUC	95% CI
ALFF_right insula/putamen	87.50%	72.22%	0.852	0.736–0.930
ALFF_left precuneus	75.00%	80.56%	0.815	0.693–0.903
fALFF_right precentral gyrus	62.50%	91.67%	0.811	0.690–0.901
fALFF_right postcentral gyrus	83.33%	72.22%	0.799	0.675–0.891
ReHo_left precuneus	87.50%	77.78%	0.847	0.731–0.927
ReHo_right SMA	75.00%	77.78%	0.782	0.657–0.879
ReHo_left fusiform gyrus	75.00%	83.33%	0.818	0.697–0.906
ReHo_right SFG	66.67%	80.56%	0.763	0.635–0.867

*SEN/SPE, sensitivity/specificity corresponding to maximum Youden index; AUC, area under the ROC curve; CI, confidence interval; SMA, supplementary motor area; SFG, superior frontal gyrus.*

### Correlation Results

**FIGURE 5:**
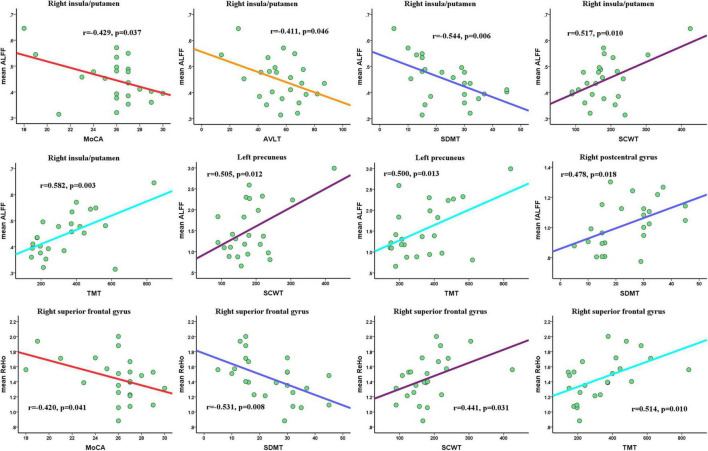
Pearson’s correlations among mean ALFF/fALFF/ReHo values of altered clusters and clinical parameters. Significance was set to *p* < 0.05. MoCA, Montreal Cognitive Assessment; AVLT, Auditory Verbal Learning Test; SDMT, Symbol Digit Modalities Test; TMT, Trail-Making Test; SCWT, Stroop Color-Word Test.

For significantly altered clusters between the CSVD-c group and the control group, we extracted the mean ALFF/fALFF/ReHo values for the CSVD-c group. As Pearson’s correlation analysis showed, the mean ALFF value of the right insula/putamen showed significantly negative correlations with MoCA (*r* = –0.429, *p* = 0.037), AVLT (*r* = –0.411, *p* = 0.046), and SDMT (*r* = –0.544, *p* = 0.006) scores and significantly positive correlations with SCWT (*r* = 0.517, *p* = 0.010) and TMT scores (*r* = 0.582, *p* = 0.003). Also, the left precuneus showed significantly positive correlations with SCWT (*r* = 0.505, *p* = 0.012) and TMT (*r* = 0.500, *p* = 0.013) scores. The mean ReHo value of the right superior frontal gyrus showed significantly negative correlations with MoCA (*r* = –0.420, *p* = 0.041) and SDMT (*r* = –0.531, *p* = 0.008) scores and significantly positive correlations with the SCWT (*r* = 0.441, *p* = 0.031) and TMT (*r* = 0.514, *p* = 0.010). For the fALFF value, only the right postcentral gyrus showed significantly positive correlations with the SDMT score (*r* = 0.478, *p* = 0.018) (shown in Figure 5).

## Discussion

ALFF/fALFF/ReHo approaches are used as effective non-invasive imaging tools to investigate intrinsic brain activities in CSVD patients. Our study is the first to utilize the combination of three methods to detect abnormal neural activities in certain brain regions in CSVD patients. One advantage of this study is that in addition to CSVD-c patients, both CSVD-n patients and healthy patients were recruited, whereas previous fMRI studies of CSVD did not recruit CSVD-n patients or healthy patients. By comparing the ALFF/fALFF/ReHo among the three groups, the present study could contribute to the exhibition of more comprehensive functional alterations.

Compared with controls, CSVD-c patients displayed increased ALFF values in the clusters of the right insula, putamen and left precuneus; decreased fALFF values in the cluster of right precentral gyrus and postcentral gyrus; and increased ReHo in the left precuneus, fusiform gyrus and right SMA, superior frontal gyrus. However, there was no significantly increased/decreased ALFF/fALFF/ReHo in these regions in CSVD-n patients, which is a notable finding. This result suggested that only CSVD-c patients had apparent changes in brain function compared with controls. A previous study suggested that leakage of the BBB is often considered to be the original mechanism of CSVD, which can lead to immune cell infiltration and inflammation (Cuadrado-Godia et al., 2018) and result in a variety of pathological processes. As one of the manifestations of CSVD, there is a close relationship between microbleeding and vascular inflammation markers. The greater burden of CMBs is often accompanied by increased markers of vascular inflammation/endothelial dysfunction and systemic inflammation (Low et al., 2019). All inflammatory markers were at higher levels in CMB patients (Miwa et al., 2011), and compared with CSVD-n patients, CSVD-c patients had more severe pathological changes. This also explains why the CSVD-c group showed elevated ALFF values compared with those of the CSVD-n group, and the changed area was similar to the changed area of the controls but slightly less. Therefore, compared with controls, only the ALFF/fALFF/ReHo values of CSVD-c patients changed significantly. CSVD-c patients showed increased ReHo in some regions compared with that of CSVD-n patients.

In the present study, the CSVD-c group displayed abnormal spontaneous neural activity associated with significantly increased ALFF values in clusters of the right insula and putamen compared with healthy controls. The human insula is divided into at least three distinct subdivisions in several FC studies. A dorsal anterior insula (DAI) region connected to the frontal, anterior cingulate, and parietal regions is relevant to cognitive control processes; a middle posterior insula subdivision with connections to brain regions is involved in sensorimotor processing; and a ventral anterior insula subdivision has connections with limbic areas for affective processes (Deen et al., 2011; Chang et al., 2013; Uddin et al., 2014). A previous study clarified that isolated putamen hemorrhage can impair frontal lobe function and cause executive dysfunction by affecting the dorsolateral-striato-pallido-thalamic circuits (Kokubo et al., 2015). When frontal lobe function is impaired, it affects cognition, emotion, behavior management, exercise and so on. In summary, both the insular lobe and putamen are closely related to cognitive and executive function. Therefore, it is reasonable for CSVD-c patients with abnormal spontaneous neural activity in these two regions to have lower MoCA, AVLT, and SDMT scores and higher SCWT and TMT scores.

Apart from increased ALFF values in the right insula and putamen, a striking difference between the CSVD-c group and the control group was found to increase ALFF values in the left precuneus. Compared with CSVD-n patients, CSVD-c patients also showed increased ALFF values. Several reviews have pointed out that the precuneus is related to higher-level cognitive functions, such as episodic memory, self-related information processing, and various aspects of consciousness; this association may be attributed to the fact that the precuneus is a central node of the default mode network (DMN) (Cavanna and Trimble, 2006). In addition, the precuneus is involved in the processing of sensorimotor information and visual information (Margulies et al., 2009). Based on the increased ALFF values in the left precuneus and the above summary of functions of the precuneus, we speculate that there will be executive function and attention changes in CSVD-c patients. The results showed that in the CSVD-c group, the higher the mean ALFF value of the left precuneus was, the higher the SCWT and TMT scores were, which confirmed our hypothesis and explained the decline in executive function and attention in CSVD-c patients. Significantly decreased fALFF values were found in the right precentral gyrus and postcentral gyrus in CSVD-c patients compared with controls. The postcentral gyrus serves as a key region in the somatosensory network, participates in daily activities (Fu et al., 2019) and governs the learning of early motor skills acquisition (Bernardi et al., 2015). The finding of decreased fALFF values in the right precentral gyrus and postcentral gyrus indicates that the integrity of the somatosensory network is compromised. Therefore, CSVD-c patients may experience reduced motor learning ability compared to the control group. This has also been confirmed in our research: the fALFF values in the right postcentral gyrus in CSVD-c patients is closely related to clinical SDMT scores.

Compared with controls/CSVD-n patients, CSVD-c patients showed increased ReHo in the clusters of left precuneus, fusiform gyrus, right SMA, superior frontal gyrus, and in the clusters of left middle frontal gyrus, superior frontal gyrus, right SMA. Although the functionality of the fusiform gyrus is not fully understood, it has been linked with various neural pathways related to recognition, mainly for facial recognition, object classification and object classification recognition. The SMA is an important part of the sensorimotor network (Fox et al., 2005; Seeley et al., 2007). The middle frontal gyrus and the superior frontal gyrus are important regions of the frontoparietal network. Several studies have demonstrated that the superior frontal gyrus has many functions, such as cognitive control and resting-state regulation (Vincent et al., 2008; Niendam et al., 2012; Andrews-Hanna et al., 2014; Raichle, 2015), working memory (Owen et al., 1996; Courtney et al., 1998; Rowe et al., 2000), and motor movement (Martino et al., 2011). Therefore, changes in the ReHo value of these areas impair related brain networks and brain functions in CSVD-c/CSVD-n patients. The above statement was also confirmed by analyzing the correlation between ReHo and clinical parameters.

In this study, for significantly altered clusters between the CSVD-c group and the control group, we extracted the mean ALFF/fALFF/ReHo values for the CSVD-c group. Compared with controls and CSVD-n patients, the more severe CSVD-c patients’ functional impairment was, the lower the MoCA, AVLT and SDMT scores were, and the higher the SCWT and TMT scores were. Notably, ALFF/ReHo outcomes were inversely correlated with MoCA, AVLT and SDMT scores and positively correlated with SCWT and TMT scores. fALFF values are directly proportional to MoCA, AVLT and SDMT scores and inversely proportional to SCWT and TMT scores. Our results showed that the increased ALFF value of the right insula/putamen cluster in the CSVD-c group was significantly related to all five clinical parameters; the sensitivity of the right insula/putamen cluster ALFF value change in the ROC curve was the highest. In addition, the increased ReHo value of the right superior frontal gyrus in the CSVD-c group was markedly correlated with four clinical parameters other than AVLT score. Therefore, we infer that the right insula, putamen and superior frontal gyrus may be three key areas that show more correlation with the severity of clinical symptoms, which are important for the exploration of neurophysiological mechanisms in CSVD-c patients.

Some limitations in this study should be considered. First, although ALFF, fALFF and ReHo are promising tools for detecting spontaneous brain functional activities, there are several factors that may influence our analysis of the results. For example, other imaging markers of CSVD, including WMHs, recent small subcortical infarcts, lacunes, PVSs, and brain atrophy, have potential effects on ALFF/fALFF and ReHo values in different regions. Second, in this study, the sample size was relatively small because the prevalence of cerebral microbleeds in normal subjects is 5–21%, which limits statistical power; therefore, the results of this study should be considered preliminary, and further research should expand the sample size to find more CSVD-c patients and verify the results. Third, this study only elaborated on the functional changes in different areas of the brain network, and follow-up studies should be conducted to explore the abnormal connections between brain functional networks.

Conclusion: In the current study, we investigated the possible pathogenesis of CSVD by analyzing resting-state spontaneous brain activity based on ALFF, fALFF and ReHo values in CSVD patients. The results suggested that abnormal changes in spontaneous brain activity in the DMN, somatosensory network, sensorimotor network, and frontoparietal network may explain the changes in clinical parameters in CSVD patients, especially in CSVD-c patients. These results expounded the underlying neurophysiological mechanisms in CSVD patients.

## Data Availability Statement

The original contributions presented in the study are included in the article/supplementary material, further inquiries can be directed to the corresponding author/s.

## Ethics Statement

The studies involving human participants were reviewed and approved by the Institutional Review Board of Shandong Provincial Hospital Affiliated to Shandong First Medical University Subcommittee on Human Studies. The patients/participants provided their written informed consent to participate in this study.

## Author Contributions

LG and CL conceived and designed the experiments. LG, CL, MF, HW, HX, and NZ performed the experiments. HW analyzed the data and created the figures. MF and HW wrote the manuscript. LG and HW provided the funding. All authors reviewed the manuscript.

## Conflict of Interest

The authors declare that the research was conducted in the absence of any commercial or financial relationships that could be construed as a potential conflict of interest.

## Publisher’s Note

All claims expressed in this article are solely those of the authors and do not necessarily represent those of their affiliated organizations, or those of the publisher, the editors and the reviewers. Any product that may be evaluated in this article, or claim that may be made by its manufacturer, is not guaranteed or endorsed by the publisher.

## Footnotes

1
www.mocatest.org

2
http://www.restfmri.net

3
https://www.medcalc.org

## Abbreviations

CSVD-c: CSVD patients with CMBs
CSVD-n: CSVD patients without CMBs
FA: fractional anisotropy
SMA: supplementary motor area
SMA.L: left SMA
ANCOVA: analysis of covariance
SPM: statistical parametric mapping
DPABI: Data Processing and Analysis for Resting-state Brain Imaging
MNI: Montreal Neurological Institute
FWHM: full width at half maximum
FFT: fast Fourier transform
KCC: Kendall coefficient of concordance
LSD: least-significant difference
GRF: Gaussian random field
MoCA: Montreal Cognitive Assessment
AVLT: Auditory Verbal Learning Test
SDMT: Symbol Digit Modalities Test
TMT: Trail-Making Test
SCWT: Stroop Color-Word Test
DAI: dorsal anterior insula
DMN: default mode network
SFG: superior frontal gyrus
^a^: ANCOVA test
TMT-A + B: sum of TMT-A and TMT-B
FD_Jenkinson: frame-wise displacement (Jenkinson et al., 2002).
